# Salicylic acid modulates arsenic toxicity by reducing its root to shoot translocation in rice (*Oryza sativa* L.)

**DOI:** 10.3389/fpls.2015.00340

**Published:** 2015-05-18

**Authors:** Amit P. Singh, Garima Dixit, Seema Mishra, Sanjay Dwivedi, Manish Tiwari, Shekhar Mallick, Vivek Pandey, Prabodh K. Trivedi, Debasis Chakrabarty, Rudra D. Tripathi

**Affiliations:** Division of Plant Ecology and Environmental Science, Department of Environmental Science, Council of Scientific and Industrial Research – National Botanical Research InstituteLucknow, India

**Keywords:** arsenate, salicylic acid, rice seedlings, antioxidants, Fe transporters

## Abstract

Arsenic (As) is posing serious health concerns in South East Asia where rice, an efficient accumulator of As, is prominent crop. Salicylic acid (SA) is an important signaling molecule and plays a crucial role in resistance against biotic and abiotic stress in plants. In present study, ameliorative effect of SA against arsenate (As^V^) toxicity has been investigated in rice (*Oryza sativa* L.). Arsenate stress hampered the plant growth in terms of root, shoots length, and biomass as well as it enhanced the level of H_2_O_2_ and MDA in dose dependent manner in shoot. Exogenous application of SA, reverted the growth, and oxidative stress caused by As^V^ and significantly decreased As translocation to the shoots. Level of As in shoot was positively correlated with the expression of *OsLsi2*, eﬄux transporter responsible for root to shoot translocation of As in the form of arsenite (As^III^). SA also overcame As^V^ induced oxidative stress and modulated the activities of antioxidant enzymes in a differential manner in shoots. As treatment hampered the translocation of Fe in the shoot which was compensated by the SA treatment. The level of Fe in root and shoot was positively correlated with the transcript level of transporters responsible for the accumulation of Fe, *OsNRAMP5*, and *OsFRDL1*, in the root and shoot, respectively. Co-application of SA was more effective than pre-treatment for reducing As accumulation as well as imposed toxicity.

## Introduction

Arsenic (As) is posing a serious health concern in South East Asia especially in Bangladesh and West Bengal in India. Long term As exposure leads to skin lesions and various types of cancers (Kumar et al., 2015). Safe level of As in drinking water is10 μg l^-1^, as recommended by World Health Organization in 1993, while the level of As in ground water has been reported up to 3200 μg l^-1^ in West Bengal and Bangladesh that is enough to show the severity of problem (McCarty et al., 2011). Arable land can be contaminated through irrigation by As rich water. More than 90% production of rice comes from South East Asia that is heavily contaminated by As, thus significant amount of As also accumulates in various parts of rice which serves as a major entry route for As in to food chain. Presence of As in grains also hampers the nutritional value of rice in terms of trace nutrients and amino acids (Kumar et al., 2014a).

Arsenic is non-essential element for plant and present in environment both in inorganic as well as organic forms. Arsenate (As^V^) and arsenite (As^III^) are predominant inorganic forms. As toxicity symptoms in plants range from inhibition of root growth, photosynthesis to death of plant (Mishra et al., 2014; Kumar et al., 2015). Arsenate shows structural analogy with phosphate so it is mainly transported through high affinity phosphate transporters (Tripathi et al., 2007). In paddy field, As^III^ is the predominant chemical species of As due to anaerobic growing conditions (Takahashi et al., 2004). Further, most of the As taken up by the plants is also reduced and stored as As^III^ (Pickering et al., 2000; Mishra et al., 2013). Arsenite is transported through aquaporin channels. Two major As^III^ transporters, Lsi1 and Lsi2 have been reported in rice. Lsi1 is localized at the distal side of both exodermis and endodermis cells of rice roots and mediates the influx of As^III^. Lsi2 is localized at the proximal side of both exodermis and endodermis cells and plays an important role in As^III^ transport to the shoots and ultimately to the rice grains (Ma et al., 2008). Arsenate can replace phosphate from many biochemical reactions leading to disruption of energy flow while As^III^ interferes with functioning of proteins and enzymes through thiol interaction (Finnegan and Chen, 2012). As is a redox active metalloid and induces the generation of reactive oxygen species (ROS) leading to lipid peroxidation, disruption of cellular redox state, and associated toxicity (Finnegan and Chen, 2012). In rice As mediated redox imbalance has been shown to the major factor causing toxicity (Srivastava et al., 2014). To cope up with ROS production plants are equipped with various antioxidant enzymes and molecules (GSH, Ascorbate). GSH also serves as substrate for phytochelatins (PCs), the metal and metalloids chelating ligands, therefore, reduces free As inside cell (Kumar et al., 2014b).

Salicylic acid and its derivative (acetylsalicylic acid) have been used for therapeutic purpose since more than a century. SA is synthesized by two pathways, the isochorismate pathway and the phenylalanine ammonia-lyase pathway (Vlot et al., 2009). SA is an important signaling molecule and its role in protection against various biotic and abiotic stresses has been well studied in plants (Yuan and Lin, 2008; Vlot et al., 2009). Upon pathogen attack endogenous level of SA gets enhanced and binds to catalase (CAT) that leads to enhanced level of H_2_O_2_. The H_2_O_2_ serves as secondary messenger to induce the expression of pathogen related proteins and ultimately initiates systemic acquired resistance (Vlot et al., 2009). SA has been reported to provide protection against heavy metal stress such as, against mercury in *Medicago sativa* (Zhou et al., 2009), cadmium stress in barley, rice and soybean (Metwally et al., 2003; Guo et al., 2007; Noriega et al., 2012), and against nickel stress in mustard (Yusuf et al., 2012). Guo et al., (2007) hypothesized that enhanced level of H_2_O_2_ by SA serves as secondary messenger to improve plant defense against abiotic stress. SA is reported to abate the chlorosis under iron deficient conditions and also promotes iron (Fe) uptake and translocation in *Arachis hypogaea* (Kong et al., 2014) and enhanced mineral nutrient uptake including Fe in maize (Gunes et al., 2007).

Iron acquisition mechanism in various plants is divided in two main categories: Strategy I in non-graminaceous plants and Strategy II in graminaceous plants (Römheld and Marschner, 1986). The two main processes in the Strategy I are the reduction of ferric chelates (Fe^+3^-chelate) at the root surface and the absorption of the generated ferrous (Fe^+2^) ions across the root plasma membrane (Kobayashi and Nishizawa, 2012). Rice belongs to family graminae which uses strategy II for Fe uptake where the plant roots secretes mugenic acid (MA) that forms Fe^+3^-MA complex and is taken up by root cells by YSL transporters (Kobayashi and Nishizawa, 2012). There are several Fe transporters in which OsFRDL1, OsYSL2, and OsNRAMP5 follow strategy II and uptake only chelated Fe^+3^, but rice also has a unique transporter OsIRT1 which enables the plant to directly uptake the Fe^+2^ from soil beyond the strategy II (Ishimaru et al., 2006). The key regulator of Fe transporters is OsIRO2, strongly induced under iron deficient conditions (Ogo et al., 2007). OsFRDL1 is expressed in rice root pericycle and encodes citrate eﬄuxer that is required for efficient Fe translocation (Yokosho et al., 2009) and OsYSL2 is responsible for long distance transport of chelated Fe^+3^ to sink tissues (Ishimaru et al., 2010). Along with the Fe^+3^, OsNRAMP5 also contributes to Mn^+2^ and Cd^+2^ transport in rice (Ishimaru et al., 2012).

This study is hypothesized to investigate positive impact of SA on As^V^ tolerance in rice. We analyzed changes in As accumulation, oxidative stress, antioxidant enzymes activities, As^III^, and Fe transporters in As^V^ exposed plants under co-application, and pre-treatment of SA.

## Materials and Methods

### Growth Conditions and Experimental Design

Seeds of *Oryza sativa* cv. Pant4 collected from Masina Research Centre, Pvt. Ltd., Bihar (India), were surface sterilized using 10% H_2_O_2_ for 30 s and washed with Milli Q water. Seeds were germinated on moist pre-sterilized blotting sheets in a tray, placed in seed germinator for 4 days at 25°C, relative humidity was 65%. After 7 days, 50 uniform size seedlings were selected and placed in 150 ml beakers, covered with black sheet, containing 100 ml of 100% Hewitt nutrient medium, prepared in Milli-Q water (pH 6.8–7.0) and grown for another 10 days under light intensity 210 μM cm^-2^s^-1^ (16/8 h; day/night). 10 days old plants were provided As^V^ (25 and 50 μM) using the salt Na_2_HAsO_4_ and SA (100 μM) in the nutrient medium and grown for 7 days. Plants treated by 25 and 50 μM As^V^, 100 μM SA for 7 days abbreviated as As^V^25, As^V^50 and SA, respectively. Plants treated with As^V^25, As^V^50 supplemented with SA abbreviated as SA + As^V^25and SA + As^V^50. For Pre-treatment of SA, plants were grown in 100 μM SA for 3 days and then transferred to Hewitt solution containing As^V^25, As^V^50for 7 days and they are abbreviated as SA Pre+As^V^25and SA Pre + As^V^50. Plants grown in As^V^ deprived medium termed as SA Pre and plants grown only in Hewitt solution served as control.

### Estimation of Chlorophyll and Carotenoids

Fresh leaves (0.1 g) were crushed in 5 ml of 80% acetone and centrifuged at 10,000 × *g* for 10 min. The supernatant was used for estimation of chlorophyll by Arnon (1949) method and carotenoids by Duxbury and Yentsch (1956) method.

### Estimation Hydrogen Peroxide and MDA

Fresh leaves (0.5 g) were crushed in 5 ml of 0.1% trichloroacetic acid and centrifuged at 10,000 × *g* for 10 min. The supernatant was used for estimation of MDA and H_2_O_2_. MDA and H_2_O_2_ by Heath and Packer (1968) and Velikova et al. (2000), respectively.

### Assay of Antioxidant Enzymes

Fresh leaves (0.3 g) were ground in liquid N_2_ using a mortar, and homogenized in 3 ml of buffer containing 50 mM potassium phosphate buffer (pH 7.8) and 1% (w/v) polyvinylpyrrolidone. The homogenate was centrifuged at 8000 × *g* at 4°C for 15 min. and supernatant was used for ascorbate peroxidase (APX), guaiacol peroxidase (GPX), CAT, superoxide dismutase (SOD), and Nitrate reductase (NR) activity, and nitrite and soluble protein concentration.

The activity of SOD (EC 1.15.1.1) was measured by Beauchamp and Fridovich (1971), APX (EC 1.11.1.11) by Nakano and Asada (1981), GPX (EC 1.11.1.7) by Kato and Shimizu (1987), CAT (EC 1.11.1.6) by Scandalios et al. (1983), NR (EC 1.7.99.4), and nitrite by Hageman and Reed (1980).

### Estimation of Non-Protein Thiolic Metabolites and Ascorbic Acid

The level of GSH and GSSG was measured by following the protocol of Hissin and Hilf (1976). Plant material (500 mg) was frozen in liquid nitrogen and homogenized in 0.1 M sodium phosphate buffer (pH 8.0) containing 25% meta-phosphoric acid. The homogenate was centrifuged at 20,000 × *g* for 20 min at 4°C. Total glutathione (GSSG and GSH) content was determined fluorometrically in the supernatant after 15 min incubation with *o*-phthaldialdehyde (OPT). Fluorescence intensity was recorded at 420 nm after excitation at 350 nm on a Hitachi F 7000 fluorescence spectrophotometer.

Non-protein thiol (NPT) content was measured by following the method of Ellman (1959). The concentration of PCs was calculated as PCs = NPT – (GSH + GSSG; Duan et al., 2011).

For estimation of ascorbic acid (Asc), fresh leaves (0.5 g) were crushed in 5 ml of 0.1% trichloroacetic acid and homogenate was centrifuged at 10,000 × *g* for 10 min. The supernatant was used for estimation of Asc by Shukla et al. (1979).

### Element Estimation

The elements (As and Fe) content was determined following Mallick et al. (2012). Briefly, plant tissues were washed three times with Milli Q water and plants separated in root and shoot and oven dried at 70°C. Dried plant tissues (root 300 and shoot 500 mg) were digested in HNO_3_: HCl (3:1). Digested samples were filtered through Whatman filter paper 42 and volume was made to 10 ml by Milli-Q water. As and Fe were estimated by using AAS (GBC Avanta S, USA) fitted with a hydride generator (MDS 2000) using NaH_2_BO_4_+NaOH (3 M) and HCl (3 M). The values were presented in μg per gram dry weight (μg g^-1^dw).

### Endogenous Salicylic Acid Estimation

Presence of SA in shoot samples were analyzed by HPLC (Dionex Ultimate 3000) using UV detector at 210 nm by following the method of Pan et al. (2010). The mobile phase was programmed with linear gradient of A (0.1% of formic acid in methanol) and B (0.1% of formic acid in water) as 0–20 min; 30–100% A, 20–22 min; 100% A and then 22–25 min; 100–30% of A. Flow rate was maintained at 0.3 ml min^-1^. Retention time for SA was recorded at 22.4 min.

### Gene Expression Analysis Using Quantitative RT-PCR

Approximately 5 μg, RNase free DNase-treated, total RNA isolated from roots of rice plants was reverse-transcribed using SuperScriptII (Fermentas, USA), following the manufacturer’s recommendation. The synthesized cDNA was diluted 1:5 in DEPC water and subjected to quantitative RT-PCR (qRT-PCR) analysis. The qRT-PCR was performed using an ABI 7500 instrument (ABI Biosystems, USA) using primers listed in Supplementary Table S1. Each qPCR reaction contained 5 μl of SYBR Green Supermix (ABI Biosystems, USA), 1 μl of the diluted cDNA reaction mixture (corresponding to 5 ng of starting amount of RNA) and 10 pM of each primer in a total reaction volume of 10 μl. The qPCR reactions were performed under following conditions: 10 min at 95°C and 40 cycles of the one step thermal cycling of 3 s at 95°C and 30 s at 60°C in a 96-well reaction plate. Actin gene was used as an internal control to estimate the relative transcript levels of the target gene. Specificity of amplicons generated in qPCR reactions was verified by melt curve analysis. Each qPCR reaction was performed in triplicate (technical replicates) for each biological replicate (three for each treatment). Relative gene expression was calculated using ^ΔΔ^CT method (Livak and Schmittgen, 2001).

### Statistical Analysis and Analytical Quality Control

The whole experiment was set up in the randomized block design. The data were subjected to Duncan’s Multiple Range Test (DMRT) for the analysis of significant difference between the treatments. Analytical data quality of the elements, was ensured through repeated analysis (*n* = 6) of Standard Reference Material. Standard Certified reference material (CRM 028-050) used for the accuracy of the AAS procured from Resource Technology Corporation, USA (Lot no. IH 028), and the values obtained varied between -3.97 to 22.86% error between ten measurements. The blanks were run all the time to eliminate the background noise.

## Results

### Morphology and Photosynthetic Pigments

Arsenate had deleterious impact on plant growth. A dose dependent decrease of 6 and 17% at 25 μM and 26 and 31% at 50 μM As^V^ was observed in root and shoot, respectively, than control. SA alone treatment enhanced the root and shoot length by 39 and 19%, respectively, than control. Co-application of SA and As^V^50 enhanced the root and shoot growth significantly (58 and 36%, respectively) than 50 μM As^V^ alone treated plants. SA pre-treated plants also experienced less toxicity during exposure to As^V^. Arsenate induced reduction in biomass was also significantly recovered by SA supplementation. Under As^V^ stress total chlorophyll was reduced significantly in dose dependent manner with maximum approximately 22% reduction at 50 μM As^V^ than control while carotenoid content was increased significantly in As^V^50 treatment than control. SA co-application with As^V^, reverted chlorophyll loss caused by As^V^ stress (**Table 1**).

**Table 1:** Effect on shoot, root lengths (cm), fresh-weight (mg), total chlorophyll (mg g^-1^fw), and carotenoid content (mg g^-1^fw) *Oryza sativa* after 7 days of treatment with different combinations of Arsenate (As^V^) and Salicylic acid (SA).

	Root length	Shoot length	Biomass	Total chl	Carotenoids
Control	3.88^cd^ ± 0.26	26.3^de^ ± 1.8	274.24^d^± 0.01	2.29^de^ ± 0.06	0.15^ab^ ± 0.008
SA	5.40^g^ ± 0.36	31.3^f^ ± 2.5	345.51^f^ ± 0.0	2.44^f^ ± 0.08	0.15^bc^ ± 0.008
As^V^25	3.63^bc^ ± 0.29	21.8^bc^ ± 1.4	226.84^ab^ ± 0.07	1.94^b^ ± 0.02	0.18^ef^ ± 0.004
As^V^50	2.85^a^ ± 0.19	18.0^a^ ± 1.2	218.94^a^ ± 0.07	1.79^a^ ± 0.07	0.18^f^ ± 0.006
SA + As^V^25	4.88^f^ ± 0.33	26.3^de^ ± 1.7	266.79^cd^ ± 0.06	2.31^e^ ± 0.01	0.14^a^ ± 0.004
SA + As^V^50	4.50e^f^ ± 0.30	24.5^cd^ ± 1.9	248.73^bcd^ ± 0.01	2.17^c^ ± 0.02	0.16^cd^ ± 0.001
SA Pre	4.28^de^ ± 0.29	28.1^e^ ± 1.9	312.84^e^ ± 0.016	2.48^f^ ± 0.05	0.16^cd^ ± 0.001
SA Pre +A s^V^25	3.52^bc^ ± 0.28	24.3^cd^ ± 1.6	253.36^bcd^ ± 0.08	2.22^cde^ ± 0.05	0.17^de^ ± 0.007
SA Pre + As^V^50	3.33^ab^ ± 0.26	20.6^ab^ ± 1.4	239.91^abc^ ± 0.01	2.19^cd^ ± 0.05	0.17^de^ ± 0.008

Values marked with same alphabets are not significantly different (DMRT, *p* < 0.05). All the values are means of three replicates ±SD.

### Element Content in Root and Shoot

The rice plants accumulated significant amount of As in upon exposure to As^V^ in dose dependant manner. In all treatments more than 90% of As was confined in to the roots. SA co-application to As^V^ treated plants had no significant impact on As accumulation in root. However, the shoot As was reduced significantly, i.e., around 30% reduction in both SA + As^V^25 and SA + As^V^50 than As^V^ alone treated plants was observed. SA pre-treated plants also accumulated 16 and 17% less As in shoot upon exposure to 25 and 50 μM As^V^, respectively, (**Table 2**).

**Table 2:** Accumulation (μg g^-1^dw) of Arsenic (As) and Fe in the root and shoot of *Oryza sativa* after 7 days of treatment with different combinations of As^V^ and SA.

	As root	As shoot	Fe root	Fe shoot
Control	–	–	364.2^a^ ± 18.1	60.2^cde^ ± 5.1
SA	–	–	403.3^a^ ± 30.0	68.3^e^ ± 7.1
As^V^25	317.82^a^ ± 66.2	28.84^bc^ ± 2.7	566.6^bc^ ± 49.2	48.4^ab^ ± 5.8
As^V^50	454.26^b^ ± 22.2	38.00^d^ ± 4.1	632.5^c^ ± 61.3	40.2^a^ ± 3.3
SA + As^V^25	290.83^a^ ± 42.9	19.96^a^ ± 2.3	522.4^b^ ± 63.1	62.8^de^ ± 3.9
SA + As^V^50	426.74^b^ ± 56.9	26.55^bc^ ± 2.9	528.8^b^ ± 41.1	58.3^cde^ ± 4.4
SA Pre	–	–	409.2^a^ ± 17.7	64.4^de^ ± 4.6
SA Pre + As^V^25	302.56^a^ ± 46.9	24.22^ab^ ± 2.7	486.6^b^ ± 38.4	55.8^bcd^ ± 5.8
SA Pre + As^V^50	432.32^b^ ± 53.3	31.19^c^ ± 1.8	551.4^b^ ± 43.6	52.3^bc^ ± 6.6

Values marked with same alphabets are not significantly different (DMRT, *p* < 0.05). All the values are means of four replicates ±SD.

Arsenate treatment significantly enhanced total Fe accumulation in comparison to control plants. However, the most of the accumulated Fe was localized in the roots. The translocation of Fe to shoot was reduced drastically (6% of total accumulation at As^V^50) in As^V^ treated plants which was 33% lower than control shoot. Co-application as well as pre-treatment of SA reduced the total Fe accumulation in comparison to As^V^ alone, however, its translocation to shoots increased significantly, i.e., 30 and 45% increased at SA + As^V^25 and SA + As^V^50 than As^V^ alone treated shoots and the level of Fe in shoots were comparable to control (**Table 2**).

### Oxidative Stress and Antioxidants

**FIGURE 1:**
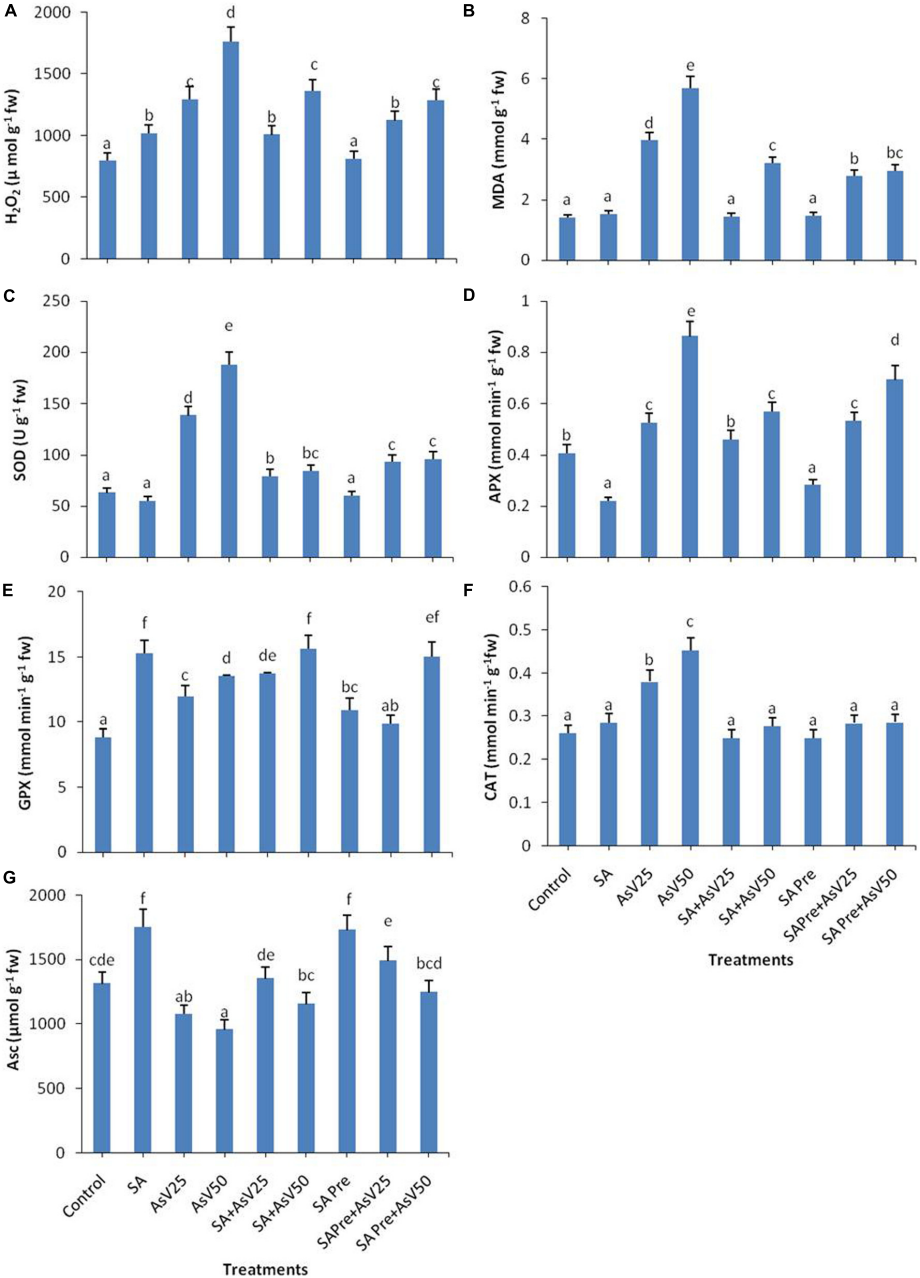
**Effect on **(A)** H_**2**_O_**2**_, **(B)** MDA, **(C)** SOD, **(D)** APX, **(E)** GPX, **(F)** CAT, and **(G)** Ascorbate in shoot of the *Oryza sativa* after 7 days of treatment with different combinations of Arsenate (As^**V**^) and Salicylic acid (SA).** Values marked with same alphabets are not significantly different (DMRT, *p* < 0.05). All the values are mean of three replicates ±SD.

Salicylic acid alone treatment had no significant impact on MDA content in rice shoot while H_2_O_2_ content was enhanced by 28% than control. Arsenate treatment enhanced the MDA content by ca. three- and fourfolds at 25 and 50 μM As^V^ exposed plants, respectively, than control. Similar trend was observed in H_2_O_2_ content. Pre-treatments as well as co-application of SA and As^V^ has reduced the level of MDA and H_2_O_2_than As^V^ alone treated plants, although co-application was more effective than pre-treatment (**Figures 1A,B**).

Salicylic acid treatment also moderated As^V^ induced antioxidant activities. SOD activity got enhanced ca. two- and threefolds, respectively, in As^V^25 and As^V^50 treated plants in shoot than control. Co-application of SA and As^V^ significantly reduced SOD activity which was about 42 and 55% than As^V^25 and As^V^50, respectively. SA pre-treatment to As^V^ exposed plants showed 32 and 50% less SOD activity the respective As^V^ treatments (**Figure 1C**).

Salicylic acid alone treatment reduced the APX activity to approximately half while 50 μM As^V^ treatment approximately doubled the APX activity than control. Co-application of SA and As^V^50 reduced APX activity by 34% also SA pre-treatment (SA Pre + As^V^50) reduced APX activity by 20% than As^V^50 treated plants (**Figure 1D**). SA alone treatment enhanced the GPX activity by ca. twofold, furthermore As^V^ treatment also enhanced the activity significantly than control. Co-application of SA and As^V^ also enhanced GPX activity than corresponding alone As^V^ treated plants (**Figure 1E**). Under As^V^ stress, CAT activity was enhanced 45 and 72% at 25 and 50 μM, respectively, than control. Co-application or pre-treatment of SA and As^V^, reduced the CAT activity than As^V^ alone exposed plants (**Figure 1F**). SA alone treatment has enhanced the Asc content by 33% while exposure to 50 μAs^V^ reduced the Asc level by upto 27% than control. Co-application of SA and As^V^ further enhanced the Asc content significantly than corresponding As^V^ alone treated plants. SA pre-treatment also enhanced the level of Asc upon As^V^ exposure in all treatments than corresponding As^V^ exposed plants (**Figure 1G**).

### Nitrate Reductase, Nitrite, and Endogenous Level of SA

**FIGURE 2:**
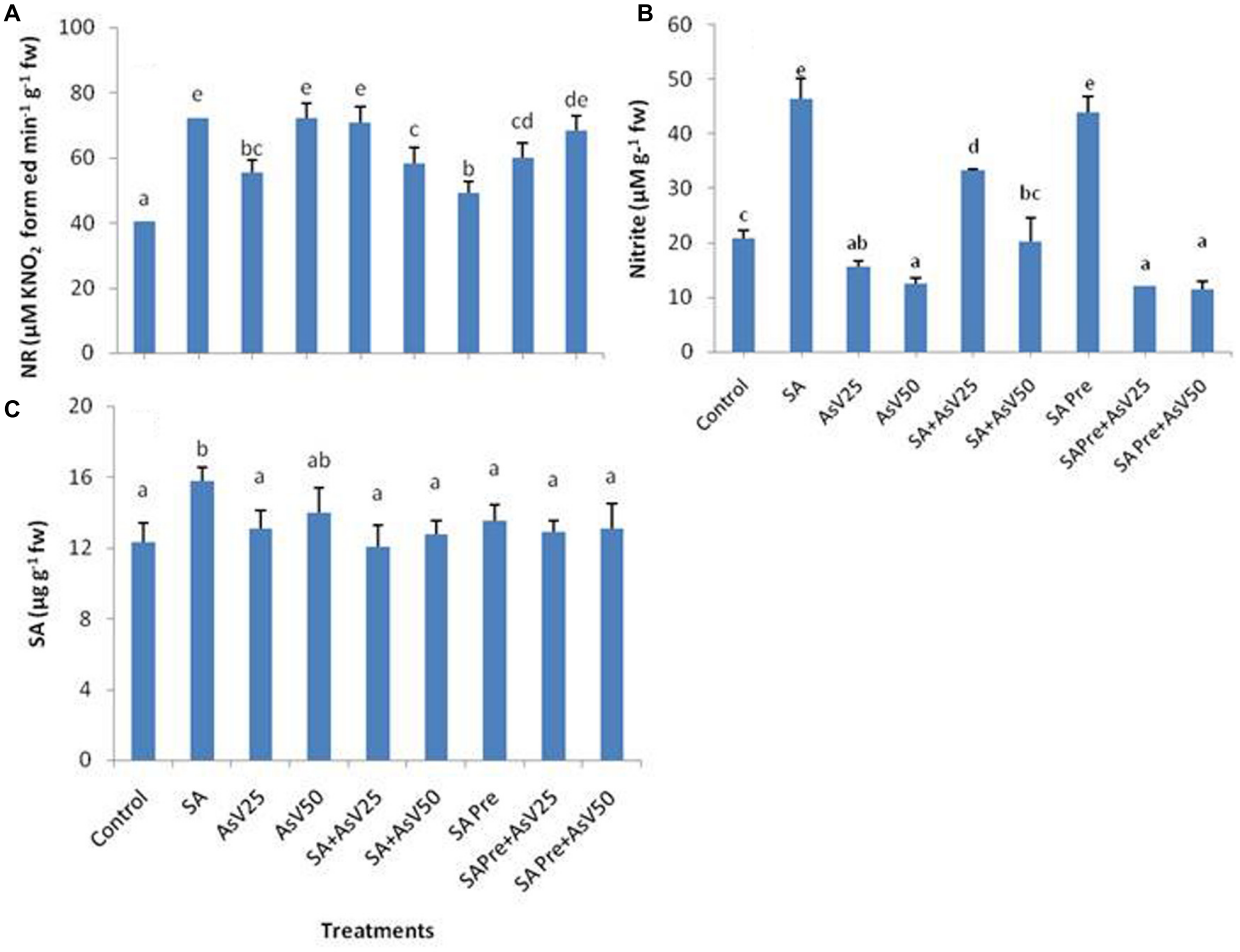
**Effect on **(A)** Nitrate reductase, **(B)** Nitrite and **(C)** Endogenous level of SA in shoot of the *Oryza sativa* after 7 days of treatment with different combinations of As^**V**^ and SA.** Values marked with same alphabets are not significantly different (DMRT, *p* < 0.05). All the values are means of three replicates ±SD.

Nitrate reductase activity was significantly enhanced by SA as well as As^V^ treated plants in comparison to control. Co-application of SA with lower As^V^ (25 μM) has enhanced the NR activity significantly while with higher As^V^ (50 μM) NR activity was reduced significantly than As alone treatments. SA pre-treatment to As^V^ exposed plants has no significant impact on NR activity than corresponding alone As^V^ exposed plants (**Figure 2A**).

The level of nitrite was almost doubled in SA alone treated plants, while a dose dependent decrease in nitrite level was observed under As^V^ stress plants than control. Co-application of SA and As^V^ enhanced the nitrite level in comparison to As^V^ alone exposed plants. SA pre-treated plants had almost double nitrite than control. However, when SA pre-treated plants were exposed to As^V^ the levels of nitrite was lower than control and were comparable to As^V^ alone treated plants (**Figure 2B**). There was no significant change in level of endogenous level of SA in shoot in all treatments except for SA alone treated plants where endogenous level of SA was enhanced significantly than control (**Figure 2C**).

### Non-Protein Thiol Metabolism

**FIGURE 3:**
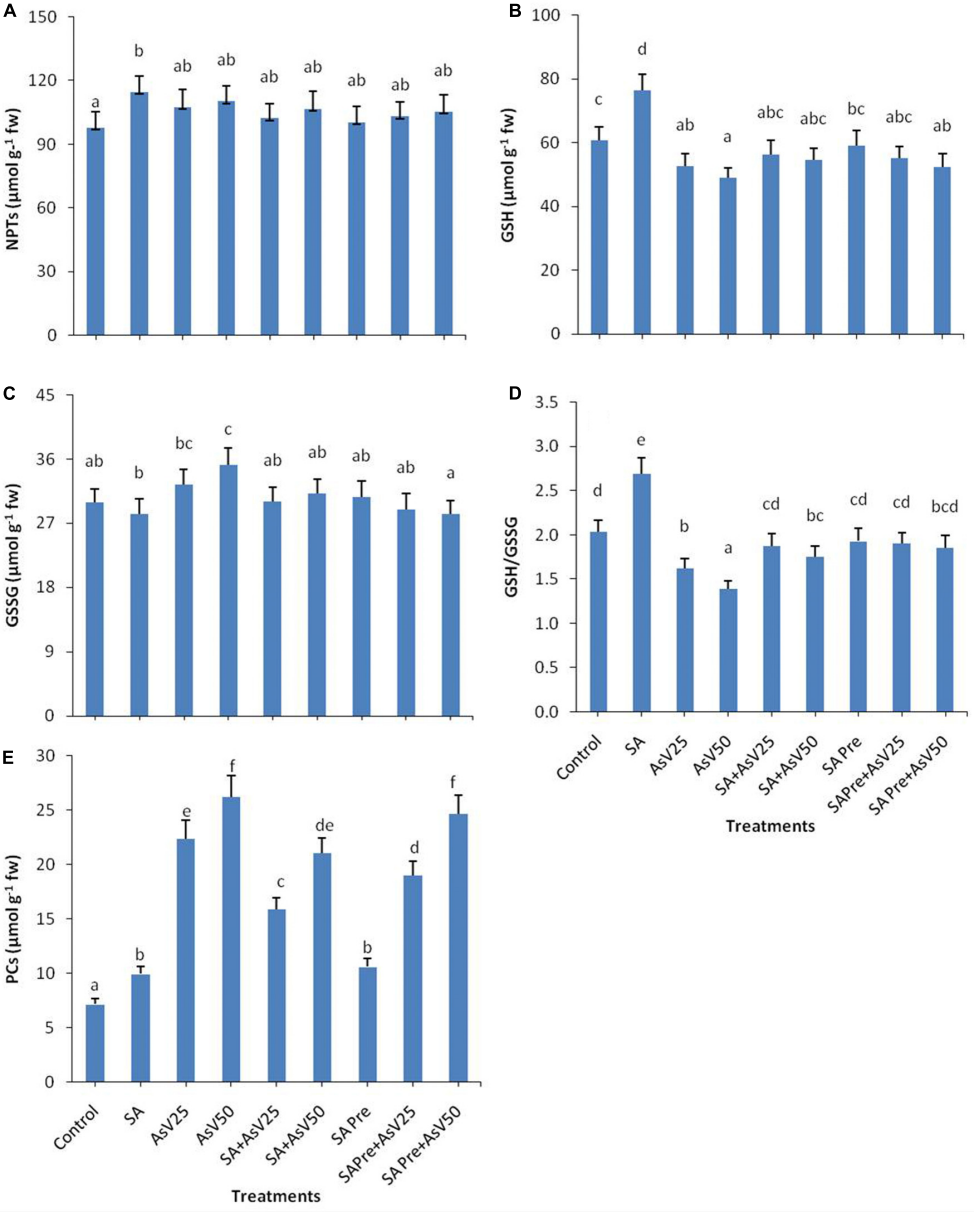
**Effect on **(A)** NPTs, **(B)** GSH, **(C)** GSSG, **(D)** ratio of GSH/GSSG, and **(E)** Phytochelatins (PCs) in shoot of the *Oryza sativa* after 7 days of treatment with different combinations of As^V^ and SA. Values marked with same alphabets are not significantly different (DMRT, *p* < 0.05).** All the values are means of three replicates ±SD.

The level of total non-protein thiol (NPT) did not show any significant change in response to the treatments in comparison to control except for SA alone treated plant (**Figure 3A**). SA treatment has enhanced the GSH level by 25% while As^V^ stress has reduced the GSH content in dose dependent manner than control. Co-application of SA and As^V^ enhanced GSH content 7 and 11% than corresponding As^V^ alone treated plants though the levels were not statistically significant different than control. Pre-treatment of SA with As^V^ had no significant impact on GSH level than corresponding As^V^ alone treated plants (**Figure 3B**). Alone SA treatment had no significant impact on GSSG level while As^V^50 has significantly enhanced GSSG content than control (**Figure 3C**). Ratio of GSH/GSSG was enhanced by 32%in SA treatment plants while in As^V^ treated plant the ratio was reduced by 20 and 31% in dose dependent manner than control. Co-application of SA and As^V^ has enhanced the GSH/GSSG ratio than their corresponding As^V^ alone treated plants. SA pre-treated As^V^ exposed plants also showed enhanced GSH/GSSG ratio than As^V^ alone treated plants (**Figure 3D**).

Both SA alone and As^V^ alone treatments enhanced the level of PCs to 1.4 and up to 3.5-fold (at As^V^50), respectively, as compared to control. Co-application of SA and As^V^ reduced the PCs accumulation by 29 and 19% than corresponding alone As^V^ treated plants though the values were still significantly higher than controls. Similar effects were observed in SA pre-treated plants both with and without As^V^ (**Figure 3E**).

### Arsenite and Iron Transporters

**FIGURE 4:**
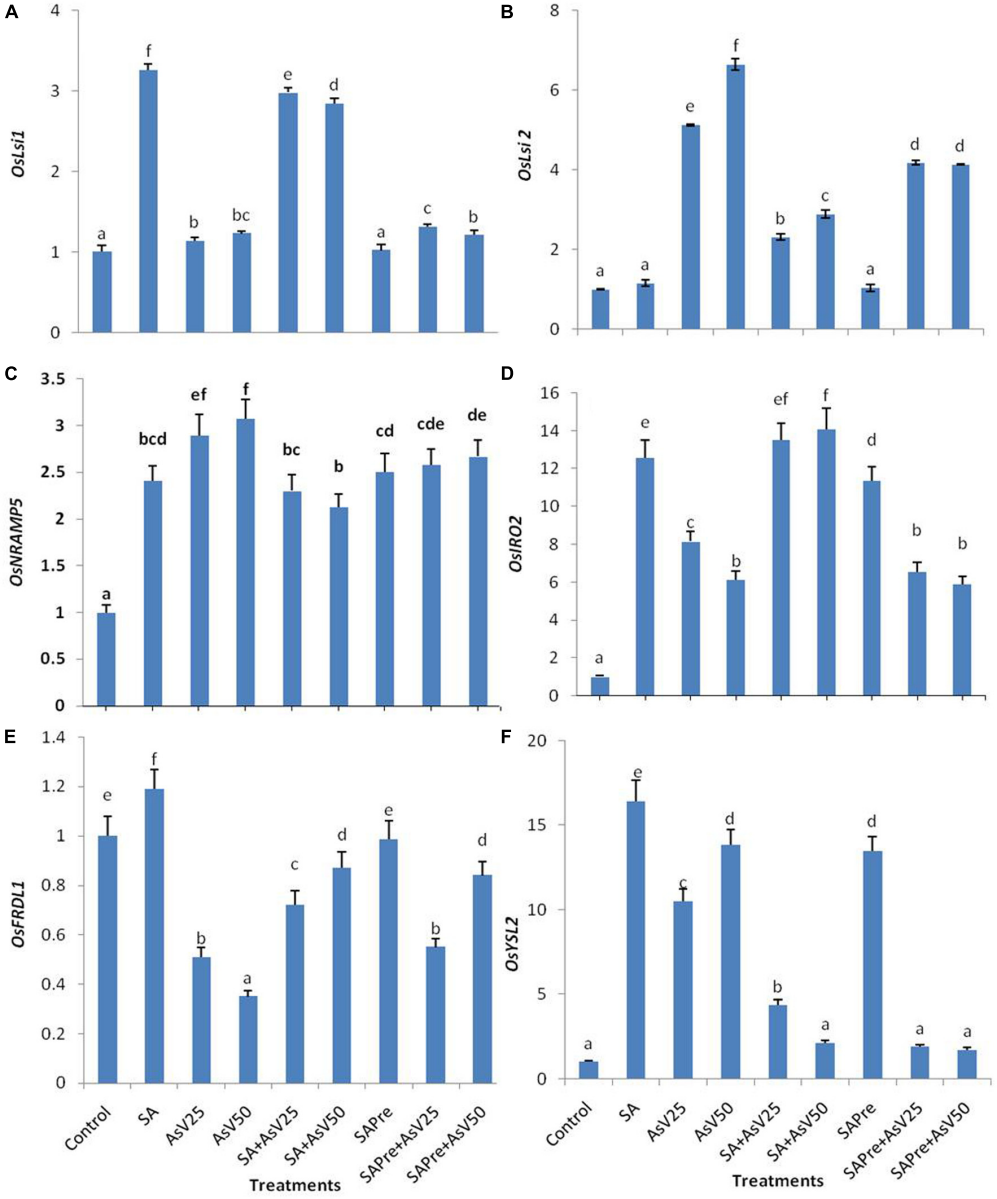
**Relative expression of transcript level of iron transporters **(A)***OsLsi1,***(B)***OsLsi2,***(C)***OsNRAMP5*, **(D)***OsIRO2*, **(E)***OsFRDL1,* and **(F)***OsYSL2* in roots of *Oryza sativa* after 7 days of treatment with different combinations of As^**V**^ and SA.** Values marked with same alphabets are not significantly different (DMRT, *p* < 0.05). All the values are means of three replicates ±SD.

Salicylic acid alone treatment enhanced the expression level of *OsLsi1* to ca. threefold than control. Arsenate alone treatment also enhanced *OsLsi1* expression significantly in comparison to control though the levels were far lower than SA alone. Co-application of SA and As^V^ enhanced *OsLsi1* expression around twofolds than As^V^ alone treated roots. Pre-treatment of SA, with or without As^V^, had no significant impact on *OsLsi1*expressionin comparison to control or respective As^V^ alone treatments (**Figure 4A**).

Arsenate exposure enhanced the *OsLsi2* expression level to ca. five- and sevenfold in dose dependent manner than control. Co-application of SA and As^V^ as well SA pre-treatment to As^V^ exposed plants lowered the expression of *OsLsi2* in comparison to As^V^ alone treatments, however, the levels were still significantly higher than control roots (**Figure 4B**).

The expression of *OsNRAMP5* was enhanced more about threefold in As^V^ treated plant roots, however, SA alone treatment also increased the expression of *OsNRAMP5* by about 2.5-folds. Co-application of SA and As^V^ has reduced the expression level by 20% and 31% in comparison to respective As^V^ alone exposed plants. SA pre-treatment slightly reduced the expression level of *OsNRAMP5* in comparison to As^V^ alone treatment which was significant at 50 μM As^V^.

*OsIRO2* expression level enhanced 13-fold in SA treated plants and As^V^25 and As^V^50 have enhanced ca. eight and ca. sixfold than control. Co-application of SA and As^V^ further enhanced expression level and that was comparable to SA treated plants. SA pre-treatment to As^V^50 stressed plants significantly increased the expression level of *OsIRO2* than As^V^50alone treated plants.

*OsFRDL1* expression level was decreased under As^V^ stress in dose dependent manner than control. Co-application of SA and As^V^ enhanced the expression level significantly than corresponding As^V^ alone treated plants. In SA pre-treated plants exposed to As^V^50 the expression level of *OsFRDL1* approximately doubled than As^V^50 alone treated plants.

*OsYSL2* expression level was enhanced to ca. 16-fold in SA alone treated plants and As^V^ alone treatment enhanced the expression by about 10- and 13-fold in dose dependent manner than control. Co-application of SA and As^V^ reduced the expression level than SA alone as well as corresponding As^V^ alone treated plants. SA pre-treatment also enhanced the expression level 13-fold than control. SA pre-treatment to As^V^ stressed plants sharply declined the expression level than corresponding alone As^V^ treated plants (**Figures 4C–F**).

## Discussions

Salicylic acid serves as an important signaling molecule in plant system which has been shown to play role in against heavy metal toxicity (as detailed in introduction). The present experiment was designed to investigate the ameliorative effect of SA during As toxicity. The co-application and pre-treatment of SA with As was used to investigate persistence of signaling aspects of SA.

Arsenic is well known to adversely affect the plant growth and development upon its accumulation (Kumar et al., 2015). In present study as well a significant amount of As was accumulated by the rice plant that hampered the plant growth severely. Application of SA, either co- or pre- treatment with As^V^ has significantly reduced the total accumulation of As (Root + shoot) with more reduction in the shoot. Though, the co-application of SA was more effective in reducing As accumulation than pre-treatment of SA. Thus, SA treatment has negatively impacted the root to shoot translocation of As. This might be due to SA-mediated down regulation of root to shoot As transporters. In present study OsLsi2, transporter responsible for root to shoot As^III^ transport in rice (Ma et al., 2008), has been found to be down regulated at mRNA level. Since As^III^ is the dominant form inside the plant (Pickering et al., 2000; Mishra et al., 2013) and also probably the main As species translocated to the shoots. Thus, down regulation of *OsLsi2* would negatively affect the As accumulation. In the present study As accumulation was positively correlation with *OsLsi2* expression level (*R* = 0.87). Down regulation of *OsLsi2*was resulted in lower As accumulation in rice shoots in response to thiourea supplementation with As (Srivastava et al., 2014). OsLsi1 is primarily responsible for As^III^ transport to root from extracellular medium, was not found correlated with root uptake of Asin present study. This might be due to fact that in present experiment plants were treated with As^V^ which is transported by the phosphate transporters (Tripathi et al., 2007). Alternatively, SA has been reported to activate ATP-binding cassette (ABC) transporters in soybean (Eichhorn et al., 2006). The ABC transporters are responsible for vacuolar sequestration of As(III)-PC complexes (Song et al., 2010). Therefore, it might be possible that most of the accumulated As in SA treated rice plants were sequestered in root vacuoles in the form of As(III)-PC, as a result less As could be transported to the shoot. Further, SA pre-treatment has been reported to enhance PCs synthesis in maize root (Szalai et al., 2013). Although SA mediated resistance against heavy metal viz., Cd, and Mn, has reported in previous studies (Metwally et al., 2003: Shi and Zhu, 2008) no reduction in the level of accumulation was observed. Since less accumulation of metalloid in shoot might also affect its level in grain which would have great implications with respect to human toxicity through food chain As contamination.

In present study SA treatment has enhanced the plant growth in terms of root, shoot length and biomass. Co-application of SA and As^V^, partially restored the plant growth in As^V^ exposed plants. Growth stimulating effects of SA has been previously reported in soybean (Gutiérrez-Coronado et al., 1998), wheat (Shakirova et al., 2003), and maize (Gunes et al., 2007). This growth restoration by SA could be auxin mediated or due to lowering of As accumulation in shoot. In SA treated wheat seedlings, higher level of auxin has been reported (Shakirova et al., 2003). SA inducible transcription factors (OBP1, OBP2, and OBP3) were found to be responsive to auxin (Kang and Singh, 2000). Pre-treatment of SA also reverted the As^V^ mediated inhibition of plant growth. In present study, a marked reduction in chlorophyll content was observed in As^V^ treated plants. Similar results were previously reported by Rahman et al. (2007) in rice and by Mishra et al. (2014) in *Ceratophyllum*. SA supplementation to As^V^ treated plants reverted As^V^ induced chlorosis. Similar reversion of chlorosis was observed in maize under salinity stress (Khodary, 2004). In present study As^V^ also reduced the Fe content in shoot that may also be responsible for As^V^ mediated chlorosis while SA has enhanced the iron content in shoot and reverted the chlorosis. Previously Kong et al. (2014) also reported the increased uptake of Fe in *Arachis hypogaea* by foliar application of SA. SA induces the nitric oxide (NO) synthesis in plants (Zottini et al., 2007) that is reported to enhance the bioavailability of Fe (Graziano and Lamattina, 2007). In present study NR activity and nitrite level was also enhanced by SA treatment, indicating enhanced level of NO that also supports above mentioned hypothesis of SA mediated enhancement of NO leading to enhanced Fe availability and increase in photosynthetic pigments.

OsFRDL1, responsible for Fe eﬄux into xylem (Inoue et al., 2004), was down regulated in As^V^ stressed plants and this decrease was concomitant with reduced Fe accumulation in shoot. OsYSL2, responsible for long distance transport of Fe (Ishimaru et al., 2010), was enhanced in both SA, and As^V^ treated plants, however, no increase in Fe accumulation in shoot was observed. OsNRAMP5 is involved in uptake of Fe in root (Ishimaru et al., 2012). The expression of OsNRAMP5 was enhanced in both SA and As^V^ treated plants and an increase in Fe accumulation in root was observed as well.

Inside the cell As induces ROS synthesis that leads to oxidative stress (Finnegan and Chen, 2012). In present study oxidative stress is indicated by enhanced level of H_2_O_2_ and MDA content in As^V^ treated plant as reported earlier in rice (Tripathi et al., 2012). Disturbed redox homeostasis in response to As^V^ has been reported as the main factor for hampered growth of rice seedlings (Srivastava et al., 2014). SA treatment reduced the level of As^V^ induced H_2_O_2_ and MDA which indicates that SA protected the plant against As^V^ mediated oxidative stress. Similar protective effects of SA has been observed against Cd stress in rice (Guo et al., 2007) and barley (Metwally et al., 2003) and against As stress in *Arabidopsis* (Odjegba, 2012). SA mediated responses are associated with H_2_O_2_ accumulation (Drazic and Mihailovic, 2005). At moderate level, H_2_O_2_ serves as a secondary messenger for activation of stress resistance mechanism in plants (Noctor and Foyer, 1998). In present study SA application activated a slight accumulation of H_2_O_2_.

Ascorbate and glutathione (GSH/GSSG) are two important antioxidants. They are redox buffering agents in the apoplast and protect the plasma membrane from oxidation (Noctor and Foyer, 1998). The ratio of GSH/ GSSG is an important marker for oxidative stress (Tausz et al., 2004). The reduced GSH/ GSSG ratio in present study showed disturbed redox balance upon As^V^ exposure. Application of SA has enhanced the GSH/ GSSG ratio. Enhanced GSH/ GSSG ratio in response to SA has also been reported in cucumber seedlings (Shi and Zhu, 2008). The enhanced As^V^ tolerance upon thiourea application was suggested to be associated with TU ability to maintain plant redox homeostasis through improved GSH/GSSG ratio (Srivastava et al., 2014). Ascorbate is an effective scavenger for free radicals (Hasanuzzaman and Fujita, 2013). Co-application of SA and As^V^ also enhanced the level of Asc thus improving the redox balance under As^V^ stress. Similar results were observed in Alfa during mercury stress (Zhou et al., 2009). The increase in the level of GSH might be due to the fact that gene encoding glutathione-dependent formaldehyde dehydrogenase /GSNO reductase was activated by SA in *Arabidopsis* (Dıìaz et al., 2003).

In present study, under As^V^ stress the activity of antioxidant enzymes APX, GPX, SOD, and CAT were enhanced in dose dependent manner. These enzymes consume the H_2_O_2_ as substrate so with the enhancement of H_2_O_2_ concentration, activity of these enzymes also enhanced. SA has high affinity to CAT and APX thereby inhibits their activities (Vlot et al., 2009; Manohar et al., 2015). In the present study SA supplementation to As^V^ stressed plants reduced the APX and CAT activity than As^V^ alone treated plants. SA is believed to inhibit CAT by the chelation of heme Fe and by causing conformational changes (Rüffer et al., 1995). However, Durner and Klessig (1996) suggested that SA-mediated inhibition of CAT probably results from peroxidative reactions.

Guaiacol peroxidase exists in various isoenzyme forms in rice and has varied functions in plant metabolism but H_2_O_2_ serves as necessary substrate for their activity. Arsenate stress enhanced the GPX activity in dose dependent manner. SA enhanced the activity of GPX as previously reported in *Medicago sativa* (Zhou et al., 2009) and in rice (Guo et al., 2007).

In present study, no significant change was observed in endogenous level of SA under As^V^ stress that is in contrast to previous studies which reported enhancement in endogenous level of SA under abiotic stress (Miura and Tada, 2014). Since rice shoot has high level of endogenous SA among all the plants tested for SA (Silverman et al., 1995), therefore, under abiotic stress endogenous level of SA may not show any significant change. Similar results were found under biotic stress when little or no change was observed in endogenous level of SA, during bacterial or fungal infection (Silverman et al., 1995; Yang et al., 2004).

## Conclusion

Taken together it is evident from present work that SA has reduced the As^V^ induced oxidative stress and effectively modulated the enzymatic and non-enzymatic antioxidants. SA also played a role in enhancing Asc, GSH, and PCs in plants subjected to As^V^ stress. SA reduced the As accumulation in shoot and also overcame the As induced Fe deficiency in shoot so the elaborated study of SA signaling may be helpful in developing the As resistant crops.

## Author Contributions

RT, PT, VP, DC, Shekhar Mallick designed experiments and reviewed manuscript. AS, GD performed experimental work and prepared figures. MT Operated Thermocycler. Seema Mishra, SD reviewed manuscript. All authors have read and approved the manuscript.

## Conflict of Interest Statement

The authors declare that the research was conducted in the absence of any commercial or financial relationships that could be construed as a potential conflict of interest.
