# Supplementary material for: Salicylic acid modulates arsenic toxicity by reducing its root to shoot translocation in rice (Oryza sativa L.)
Source: Front Plant Sci. 2015 May 18;6:340. doi: 10.3389/fpls.2015.00340 (PMC4434920; doi:10.3389/fpls.2015.00340)
Supplement: Supplementary file 1 [file Table_1.DOCX]

**Supplementary Table S1** Primer sequences of Arsenite and Iron transporters

| **Gene** | **Oligonucleotide** |
| --- | --- |
| *OsLsi1F* | GACTTCTTCCCTCCTCACCT |
| *OsLsi1R* | GCCGACGGCGTAGATCATCA |
| *OsLsi2F* | ATGAGTGAGCTTGCGTCGG |
| *OsLsi2R* | CAGGATTGGGAGGTCGATGGA |
| *OsIRO2F* | AAGCTCTACTCCTCCCTCCG |
| *OsIRO2R* | CTTCTGCAGCTCGGGTATGT |
| *OsNRAMP5F* | AAGAGGACGCCGACAAGTG |
| *OsNRAMP5R* | TGTGCCGGTAATAGTGGAGC |
| *OsFRDL1F* | GGCATTCCTTTTGTCGCTGG |
| *OsFRDL1R* | AGATAGCAGCCACACCAACC |
| *OsYSL2F* | TGTCTTGAGCTGTCTGCTGG |
| *OsYSL2R* | ATCCCTGTATTTGCGTGGCA |
| Rice actin F | GGAAGTACAGTGTCTGGATTGGAG |
| Rice actin R | TCTTGGCTTAGCATTCTTGGGT |
